# First Investigation of the Spring Dietary Composition of Siberian Musk Deer (*Moschus moschiferus*) Using Next-Generation Sequencing

**DOI:** 10.3390/ani14243662

**Published:** 2024-12-18

**Authors:** Nari Kim, Areum Kim, Je-Min Lee, Ah-Young Kim, Yujin Lee, Yeonghoon Jo, Kiyoon Kim, Kyung-Hyo Do, Kwang-Won Seo, Kwang-Bae Yoon, Dong-Hyuk Jeong

**Affiliations:** 1 College of Veterinary Medicine, Chungbuk National University, Cheongju 28644, Republic of Korea; queng91@gmail.com (N.K.); gine9729@naver.com (Y.L.); wildwildjo@chungbuk.ac.kr (Y.J.); anikest@naver.com (K.K.); pollic@chungbuk.ac.kr (K.-H.D.); vetskw16@cbnu.ac.kr (K.-W.S.); 2 Center of Endangered Species, National Institute of Ecology, Yeongyang 36531, Republic of Korea; arkim@nie.re.kr (A.K.); cmlee@nie.re.kr (J.-M.L.); didud333@nie.re.kr (A.-Y.K.); 3 Wildlife Center of Chungbuk, Cheongju 28116, Republic of Korea

**Keywords:** ungulate, diet, conservation, ecology, DNA metabarcoding, fecal analysis

## Abstract

Understanding the dietary composition of the Siberian musk deer (*Moschus moschiferus*) is critical for informing conservation efforts, particularly given the species’ vulnerable status in the Republic of Korea and its limited ecological data. Previous dietary studies have relied on conventional methods with limitations in taxonomic precision, hindering comprehensive insights into their feeding ecology. To address this gap, we used Next-Generation Sequencing to analyze 16 fecal samples collected in April from Gangwon Province, the habitat of *M. moschiferus*. Our results revealed 213 unique plant species, with *Morus*, *Quercus*, and *Prunus* accounting for the majority of the diet. Notably, nearly half of the identified genera, including *Morus*, *Actinidia*, and *Viscum*, were reported for the first time as dietary plants of *M. moschiferus*. This study confirms that *M. moschiferus* is a concentrate feeder that primarily relies on woody plants (98.8%) and may exhibit tree-climbing behavior to access parasitic shrubs like *Viscum*. These findings provide a foundation for future dietary analysis research and serve as valuable baseline data for the long-term conservation of *M. moschiferus*.

## Simple Summary

Siberian musk deer (*Moschus moschiefrus*) is a vulnerable species on the IUCN Red list, facing threats from illegal poaching and habitat loss. Understanding its dietary habits and ecological needs is essential for supporting conservation efforts. This study is the first to use Next-Generation Sequencing to analyze the diet of *M. moschiferus*. Our results revealed that the species primarily fed on woody plants in April, with *Morus* and *Quercus* being the most consumed genera. Almost half of the identified genera had not previously been reported as part of the *M. moschiferus* diet using conventional methods, highlighting the need for DNA metabarcoding to more accurately compare the dietary composition of *M. moschiferus* across different regions. This research provides valuable insights into the types of plants *M. moschiferus* may consume during the spring season.

## 1. Introduction

The Siberian musk deer (*Moschus moschiferus*) is a solitary ungulate belonging to the family Moschidae, which also includes alpine musk deer (*Moschus chrysogaster*), Anhui musk deer (*Moschus anhuiensis*), black musk deer (*Moschus fuscus*), forest musk deer (*Moschus berezovskii*), Himalayan musk deer (*Moschus leucogaster*), and Kashmir musk deer (*Moschus cupreus*). The genus *Moschus* is naturally found across Asia, ranging from Afghanistan to Siberia, in mountainous regions [1]. Among them, *M. moschiferus* is the most widely distributed species in China, Russia, Mongolia, Kazakhstan, and Korea [2,3,4]. Owing to illegal poaching for musk and habitat fragmentation, *M. moschiferus* is classified as vulnerable on the IUCN Red List [5].

In the Republic of Korea (Korea), *M. moschiferus* is classified as a Class I endangered species by the Korean Ministry of Environment [6,7] and designated as Natural Monument No. 216 by the Cultural Heritage Administration of Korea. The exact population size of *M. moschiferus* remains unknown, and recent camera trapping efforts have revealed that its habitat is limited to Gangwon Province. Despite the critical need for species conservation, obtaining information is severely restricted due to the small population size and the species’ tendency to inhabit rugged mountainous terrain [7].

The musk deer is recognized as a concentrate selector, feeding mainly on the foliage of trees, shrubs, and forbs because of its relatively small body size compared to that of other ruminants [8,9,10,11]. The energy requirement of this small ruminant is high because of its higher basal metabolic rate per unit of body weight compared to larger ruminants, necessitating the selection of high-quality forage [12,13,14]. Several studies have investigated the dietary composition of wild musk deer using fecal analysis with microscopic techniques, forage station investigations, and gastric content analyses. *M. moschiferus* has been reported to feed on up to 84 different plant species during winter in China [15,16,17] and 64 in Russia [18], demonstrating its ability to endure periods of food scarcity [19]. Meanwhile, *M. leucogaster* in India consumed a high proportion of forbs and woody leaves during autumn and winter, while forbs and lichens predominated during spring and summer [8]. Similarly, another study reported an increased proportion of browse (woody plants), particularly *Pinus* species, along with a decreased availability of graminoids (grass and sedges families) and forbs (broad-leaved herbaceous plants) in the winter diet of *M. leucogaster* [20]. However, these findings were based on conventional methods with several limitations, including low precision in identifying degraded food sources, underestimation of masticated diets, and the requirement for expert knowledge [21].

Recent developments in DNA-based approaches have enabled more accurate dietary assessments through Next-Generation Sequencing (NGS) [22]. This technology has been widely applied in the dietary studies of various wildlife species, including the Pyrenean desman (*Galemys pyrenaicus*), Korean water deer (*Hydropotes inermis argyropus*), Eurasian otter (*Lutra lutra*), and Tricolor langur (*Presbytis chrysomelas cruciger*) [23,24,25,26]. By allowing higher taxonomic precision with a small amount of feces [27], DNA metabarcoding is particularly suitable for analyzing the diet of endangered species. Despite technical challenges and limited sequencing databases, NGS-based diet analysis is expected to gain broader use for its high data abundance and sensitivity in detecting rarely consumed items [28].

Understanding dietary habits and feeding ecology is crucial to effectively conserve endangered wildlife. Several studies have demonstrated that molecular dietary analyses can support the development of effective, data-driven conservation strategies for endangered species [29,30,31]. Research on *M. moschiferus* is minimal, making analysis of its food sources essential for gaining insights into its feeding habits and informing its ecological conservation. In this study, we used NGS technology to identify the dietary plants of wild *M. moschiferus* in Korea. This study represents the first attempt to analyze the dietary composition of musk deer using NGS, aiming to highlight the potential of DNA metabarcoding as a tool for dietary analysis in *M. moschiferus*.

## 2. Materials and Methods

### 2.1. Sample Collection

**Figure 1:**
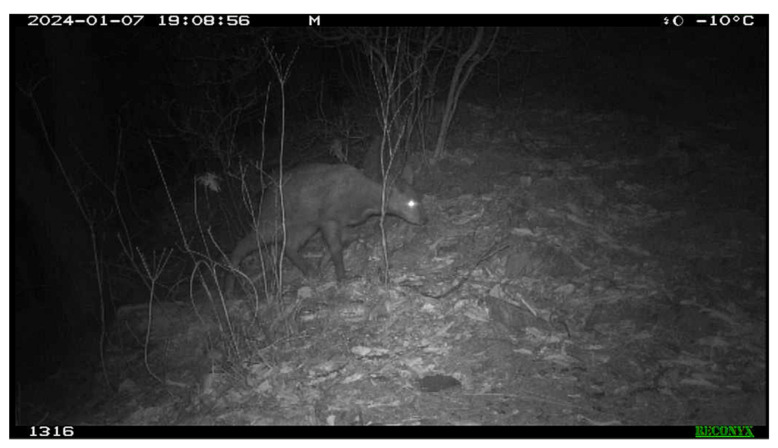
Siberian musk deer (*Moschus moschiferus*) captured by a camera trap in the study area. Image courtesy of the National Institute of Ecology.

**Figure 2:**
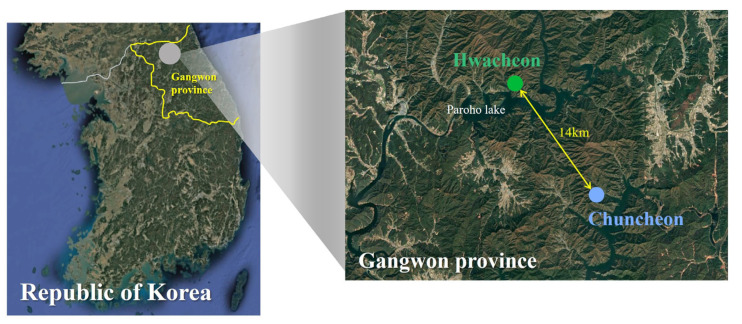
Sampling sites in Ganwon province for the collection of wild *M. moschiferus* fecal samples.

Unmanned sensor cameras were used to identify the habitat areas of *M. moschiferus* in Chuncheon and Hwacheon, Gangwon Province (Figure 1). Sixteen fecal samples were collected in April 2024, with eight samples from Chuncheon and eight from Hwacheon, located approximately 14 km apart (Figure 2). Considering that the home range of male *M. moschiferus* spans 1 km^2^ [32], fecal samples were collected from a minimum of 1 km apart. Sampling was conducted at least once a week, and only fresh, moist, and sticky fecal samples were preserved. They were stored in a deep freezer at −80 °C for one month until DNA extraction.

### 2.2. Study Area

The sampling sites were located at altitudes of approximately 650 m in Chuncheon (37.8816° N, 127.7291° E) and 430 m in Hwacheon (38.1052° N, 127.7067° E). The average precipitation and mean temperature in the sampling sites during April 2024 were 24.7 mm and 14.6 °C, respectively, according to data from the nearest meteorological observation centers of the Korean Meteorological Administration. The vegetation in these areas primarily consisted of mixed forests, including broad-leaved trees (e.g., oaks), coniferous trees (e.g., pines), and shrubs (e.g., azaleas), with no differences in vegetation profiles observed between the two sampling sites. Sympatric ungulate species of *M. moschiferus*, such as water deer (*H. inermis argyropus*), roe deer (*Capreolus pygargus bedfordi*), long-tailed goral (*Naemorhedus caudatus*), and wild boar (*Sus scrofa*), were identified through unmanned sensor camera footage (Figure S1). The leopard cat (*Prionailurus bengalensis*) and yellow-throated marten (*Martes flavigula*) were observed and identified as potential predators of *M. moschiferus* cubs.

### 2.3. DNA Extraction and Sequencing

Fecal samples (150 mg) were transferred into conical tubes, with the inner portions sliced and used under sterile conditions to minimize potential contamination. Preliminary homogenization was performed manually with 4 mL of ultra-pure water, followed by bead homogenization using a bead beater (Omni International, Kennesaw, GA, USA). Genetic identification was conducted to confirm that the fecal samples originated from *M. moschiferus*. Total genomic DNA was extracted using the Puregene Cell Kit and Tissue Kit (Qiagen, Venlo, The Netherlands), and partial mitochondrial DNA sequences were amplified using polymerase chain reaction (PCR) amplification with species-specific primers (MuskcytF: TCGGCTCGCTAATAGGCATC; MuskcytR: GCCTCGTCCTACCTGTATAAAC). The obtained sequences were subjected to Basic Local Alignment Search Tool (BLAST), version 2.15.0, against the National Center for Biotechnology Information (NCBI) GenBank database for species identification of *M. msochiferus*.

After confirming that the fecal samples were from *M. moschiferus*, the DNeasy PowerSoil Pro Kit (Qiagen) was used for plant DNA metabarcoding. The quality of the extracted DNA was assessed using a Qubit fluorometer (Thermo Fisher Scientific, Waltham, MA, USA). Unique plant-specific internal transcribed spacer (*ITS*) primer pairs, *ITS*-p3 and *ITS*-u4, were employed for amplicon sequencing of plant DNA [33]. Following PCR, library amplification was performed using the Nextra XT Index Kit to attach indices (Illumina, San Diego, CA, USA). The library quality was assessed using a Qubit fluorometer (Thermo Fisher Scientfic). The libraries were quantified, normalized, and pooled using the MiSeq Reagent Kit V3 (Illumina), and NGS was conducted on the Illumina MiSeq platform (600 cycles).

### 2.4. Operational Taxonomic Unit and Taxonomy Assignments

The resulting *ITS* gene sequences in FASTQ format were filtered and processed using QIIME2 version 2023.7 (https://qiime2.org, accessed on 2 July 2024). Zero-radius operational taxonomic unit (ZOTU) data were generated, followed by demultiplexing, trimming, merging, and denoising using a deblur algorithm. A plant reference database specific to plant *ITS*2, DB4Q2, was assigned to each ZOTU using QIIME2 [34]. Stacked bar charts and a table illustrating the relative abundances of the identified plants were created using R version 4.2.3 (https://www.r-project.org, accessed on 22 July 2024). Stacked bar charts displayed the relative abundance of dietary composition in *M. moschiferus* at the order, family, and genus levels. Species-level identification was excluded from the analysis due to the low taxonomic resolution of *ITS* target sequencing. Additionally, the table categorized the detected plant genera by their growth form: woody, forb, and graminoid [24]. Woody plants were further classified into tree, shrub, parasitic shrub, and vine, with shrubs typically defined as plants less than 3 m tall. Due to the limited sample size and the similarity of vegetation between the study areas, diversity analyses were not conducted.

## 3. Results and Discussion

**Figure 3:**
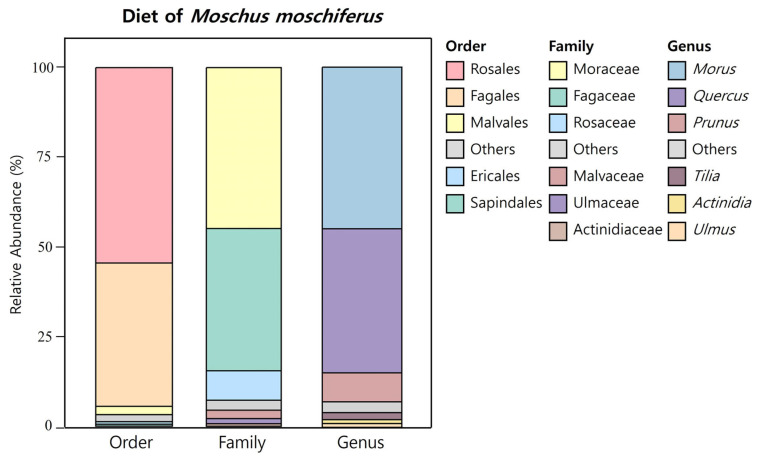
Relative abundance of diet composition in *M. moschiferus* at the order, family, and genus levels. Stacked bar charts represent the composition with a mean relative abundance of more than 0.5%.

Sequencing of the *ITS* gene libraries from 16 fecal samples of *M. moschiferus* generated 321,946 sequence reads and 213 ZOTUs. These ZOTUs were classified into 1 phylum, 5 classes, 19 orders, 31 families, and 35 genera, with 0.6% of the sequences remaining unclassified at the genus level. Streptophyta was the only phylum detected, and most of the identified classes belonged to Magnoliopsida (99.23%). Figure 3 presents bar charts illustrating the relative abundances of dietary components at the order, family, and genus levels. The dominant orders were Rosales (54.5%), Fagales (40.1%), Malvales (2.2%), Ericales (0.7%), and Sapindales (0.5%). At the family level, Moraceae (44.9%) and Fagaceae (39.7%) were most abundant, followed by Rosaceae (8.2%), Malvaceae (2.2%), Ulmaceae (1.4%), and Actinidiaceae (0.7%). At the genus level, *Morus* (44.9%) and *Quercus* (39.7%) comprised the majority of the diet (84.7%). Other genera identified in the diets included *Prunus* (7.8%), *Tilia* (2.2%), *Ulmus* (1.4%), and *Actinidia* (0.7%).

When the identified plants were categorized according to their growth form, most genera were classified as woody plants (98.8%), including trees, shrubs, parasitic shrubs, and vines (Table 1). The trees (*Morus*, *Quercus*, *Tilia*, *Ulmus*, *Actinidia*, *Acer*, *Pinus*, *Betula*, and *Juglans*) represented both deciduous and coniferous species (89.21%), with the majority being deciduous, except for *Pinus*. *Rubus* were categorized as shrubs (0.32%) and were typically less than 3 m tall. *Prunus*, *Clerodendrum*, and *Alnus* had both tree and shrub growth forms (8.22%). *Viscum* was categorized as a parasitic shrub (0.25%), growing on various types of trees. *Actinidia* and *Pueraria* spp. were found on the vines (0.84%). Another growth form, moss, was identified and represented by *Chionoloma* (0.28%).

Overall, we successfully generated 213 ZOTUs, classified them into 35 genera, and identified more than 99% of the total sequences. Notably, nearly half of the genera, including *Morus*, *Actinidia*, *Chionoloma*, *Rubus*, *Viscum*, *Clerodendrum*, *Alnus*, and *Pueraria*, were identified for the first time as dietary plants of *M. moschiferus* in our study. These genera have not been detected in previous dietary studies on *M. moschiferus* in winter in China and Russia using conventional methods [15,16,17]. These discrepancies may result from several factors, including differences in habitats, seasonal variations, and methodological approaches. Specifically, the use of different dietary analysis methods has made direct comparisons challenging. Our study provides the first comprehensive DNA metabarcoding-based dietary analysis of *M. moschiferus*, offering a more accurate and reliable assessment of its diet. Future research employing DNA metabarcoding to analyze the dietary composition of *M. moschiferus* across Korea, China, and Russia during various seasons could provide a more comprehensive and accurate assessment.

More than 98% of the identified plant genera were classified as woody plants, confirming that *M. moschiferus* is a true concentrate feeder. Since *M. moschiferus* inhabits forested alpine regions, it likely primarily feeds on woody plants, such as browsed and fallen leaves. This finding aligns with the vegetation survey, which predominantly identified broad-leaved trees and coniferous trees. Since no previous studies have investigated the relative proportions of dietary sources for *M. moschiferus*, we compared our results with a DNA metabarcoding dietary study of water deer—a sympatric ungulate species—in Korea [24]. The dietary patterns observed in Korean water deer, another concentrate selector, were consistent with those of *M. moschiferus,* with woody plants being the main dietary source (84.6%) other than graminoid and forb in the forest area. Among the food sources of *M. moschiferus*, *Morus* (44.94%), *Quercus* (39.74%), and *Prunus* (7.83%) were the most abundant, with *Morus* also being the genus most eaten by water deer. *Quercus* and *Prunus* are specifically consumed by water deer living in forests, suggesting that these genera of woody plants are key food sources in forested environments in Korea. *Tilia*, *Ulmus*, *Viscum*, *Alnus*, and *Juglans* were absent from the diet of the water deer, possibly due to regional and vegetation differences. For a more accurate comparison of dietary composition among sympatric species and *M. moschiferus*, dietary analyses using fecal samples from the same regions should be conducted.

*Viscum* is a parasitic shrub that grows on the branches of trees such as *Morus*, making it difficult to access without climbing. Interestingly, this suggests that *M. moschiferus* may climb trees to obtain *Viscum*—an unusual ruminant behavior. The alpine musk deer has been reported to have the ability to leap into *Ulmus* trees as tall as 3 m and feed on their leaves [35]. The elongated and muscular hind limbs of musk deer not only facilitate movement in snowy mountains [7] but also aid in climbing trees, enhancing its survival during times of food scarcity. Although mosses have also been identified as part of the dietary composition, the presence of *Chionoloma* in Korea remains uncertain, raising the possibility that other genera within the Pottiaceae family may have been misidentified. A detailed investigation of moss species near the sampling sites could help to accurately identify the specific genera involved.

This study has several limitations due to the small sample size, short sampling duration, and restricted vegetation surveys. The absence of other plant types such as forb and graminoid in our results may be influenced by the number and timing of the sampling, as some areas in Gangwon Province were still covered in snow during April, potentially restricting feeding options to woody plants. A more comprehensive, long-term study across seasons, along with detailed vegetation analysis, is essential to gain a clearer understanding of the dietary composition of *M. moschiferus*. Nevertheless, our study provides important information on the feeding preferences and unique dietary characteristics of *M. moschiferus* in Korea. Given the estimated population of fewer than 40 individuals in Korea [36] and the elusive nature of the species, which complicates direct observations, analyzing feeding patterns through fecal DNA metabarcoding is essential for developing effective conservation strategies. Using this dietary information, management and conservation plans could focus on protecting habitats where the most frequently consumed plant species are found.

## 4. Conclusions

This study represents the first DNA metabarcoding dietary analysis of musk deer, specifically *M. moschiferus*, a vulnerable ungulate species. *M. moschiferus* was found to be a browser that primarily consumed *Morus*, *Quercus*, and *Prunus* as the key plant genera. These findings underscore their reliance on woody plants, and the presence of *Viscum* suggests possible tree-climbing behavior to access hard-to-reach food sources. These insights are crucial for understanding the ecological needs of *M. moschiferus* and for guiding conservation management efforts. Further research, including different seasons and vegetation surveys, will provide a more comprehensive understanding of dietary composition. This dietary information can help inform habitat management by ensuring that *M. moschiferus* has access to the right plants, supporting their population and contributing to the sustainability of forest habitats.

## Figures and Tables

**Table 1:** Order, family, and genus of plants identified in the spring feces of *M. moschiferus*, along with their relative abundance and growth form. Only taxa with a mean relative abundance of more than 0.05% are shown.

Order	Family	Genus	Relative Abundance (%)	Growth Form	
Rosales	Moraceae	*Morus*	44.94	Woody	Tree
Fagales	Fagaceae	*Quercus*	39.74	Woody	Tree
Rosales	Rosaceae	*Prunus*	7.83	Woody	Tree/Shrub *
Malavales	Malvaceae	*Tilia*	2.19	Woody	Tree
Rosales	Ulmaceae	*Ulmus*	1.38	Woody	Tree
Ericales	Actinidiaceae	*Actinidia*	0.71	Woody	Vine
Sapindales	Sapindaceae	*Acer*	0.40	Woody	Tree
Pinales	Pinaceae	*Pinus*	0.39	Woody	Tree
Pottiales	Pottiaceae	*Chionoloma*	0.28	Mosses	-
Rosales	Rosaceae	*Rubus*	0.32	Woody	Shrub
Santalales	Viscaceae	*Viscum*	0.25	Woody	Parasitic shrub
Lamiales	Lamiaceae	*Clerodendrum*	0.21	Woody	Tree/Shrub *
Fagales	Betulaceae	*Alnus*	0.17	Woody	Tree/Shrub *
Fabales	Fabaceae	*Pueraria*	0.13	Woody	Vine
Fagales	Betulaceae	*Betula*	0.11	Woody	Tree
Fagales	Juglandaceae	*Juglans*	0.05	Woody	Tree

* Denotes genera that include both trees and shrubs (typically less than 3 m tall).

## Data Availability

The data presented in this study are available upon request from the corresponding author due to privacy concerns related to the geographic location.

## Supplementary Materials

The following supporting information can be downloaded at https://www.mdpi.com/article/10.3390/ani14243662/s1: Figure S1: Sympatric ungulate species of *M. moschiferus* in the study area. Image courtesy of the National Institute of Ecology. (A) Korean water deer (*H. inermis argyropus*). (B) Long-tailed goral (*Naemorhedus caudatus*). (C) Roe deer (*Capreolus pygargus bedfordi*). (D) Wild boar (*Sus scrofa*).
