# Supplementary material for: First Investigation of the Spring Dietary Composition of Siberian Musk Deer (Moschus moschiferus) Using Next-Generation Sequencing
Source: Animals (Basel). 2024 Dec 18;14(24):3662. doi: 10.3390/ani14243662 (PMC11672623; doi:10.3390/ani14243662)
Supplement: Supplementary file 1 [file animals-14-03662-s001.zip › animals-3337399-supplementary.pdf]

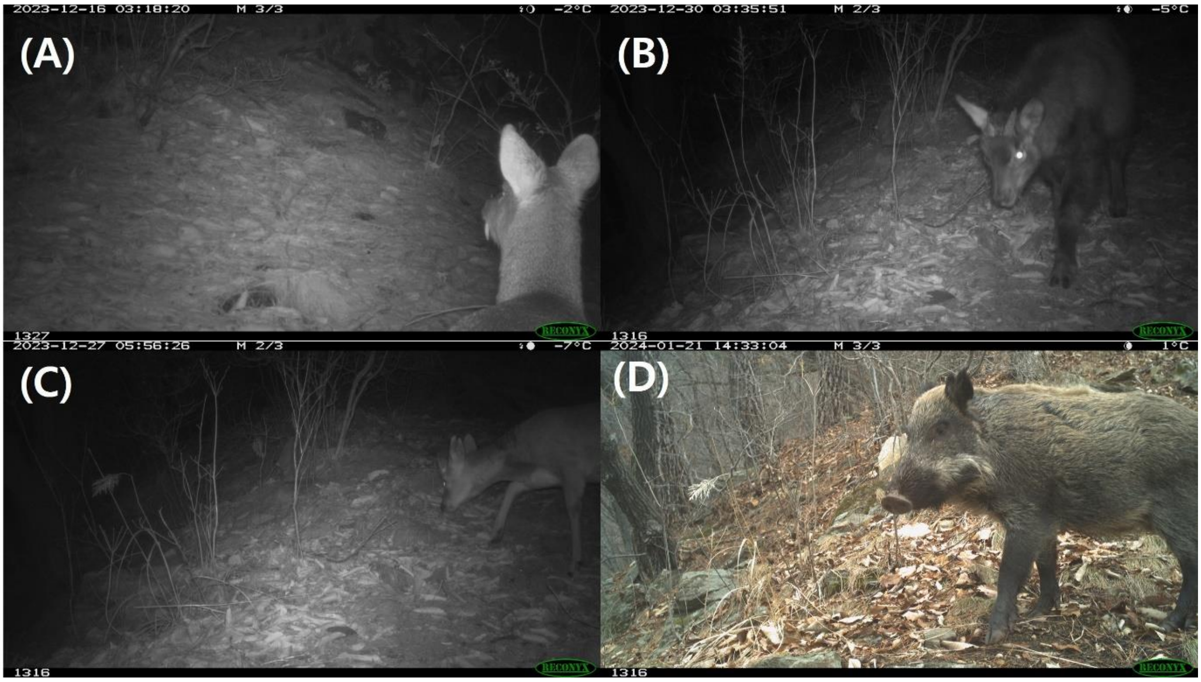

Supplementary Figure S1. Sympatric ungulate species of *M. moschiferus* in the study area. Image courtesy of the National Institute of Ecology. (A) Korean water deer (*H. inermis argyropus*). (B) Long-tailed goral (*Naemorhedus caudatus*). (C) Roe deer (*Capreolus pygargus bedfordi*). (D) Wild boar (*Sus scrofa*).
